# The effect of miR-539 regulating TRIAP1 on the apoptosis, proliferation, migration and invasion of osteosarcoma cells

**DOI:** 10.1186/s12935-021-01909-9

**Published:** 2021-04-20

**Authors:** Huowen Liu, Min Yang, Yufeng Zhang, Zhiqiang Yang, Zhe Chen, Yuanlong Xie, Binglong Peng, Lin Cai

**Affiliations:** 1 Department of Spine Surgery and Musculoskeletal Tumor, Zhongnan Hospital of Wuhan University, Wuhan, People’s Republic of China; 2 Department of Joint Surgery, Jiangxi Pingxiang People’s Hospital, Pingxiang, People’s Republic of China

**Keywords:** miRNA-539, TRIAP1, Osteosarcoma, Cell proliferation

## Abstract

**Objective:**

The purpose of this study is to explore the effect of miRNA-539 on osteosarcoma (OS) and the underlying mechanism, so as to find a new method for early diagnosis and treatment of osteosarcoma.

**Method:**

miRNA-539 mimics was transfected into osteosarcoma cells 143b and MG-63 and upregulated the expression of miR-539. QT-PCR was used to detect transfection efficacy. CCK-8 method was used to detect proliferation of 143b and MG-63 osteosarcoma cells and flow cytometry was used to detect the apoptosis of osteosarcoma cells 143b and MG-63. Wound-healing test and Transwell test were used to detect the migration and invasion ability of osteosarcoma cells. TRIAP1 was found to be the potential target gene of miRNA-539 by online bioinformatics software and the expression level of TRIAP1 in osteosarcoma cells overexpressing miRNA-539 was detected by qT-PCR. Western blot was used to detect the level of expression of TRIAP1 and its downstream genes (p53, p21, apaf1 and caspase9) in osteosarcoma cells 143b and MG63 transfected with miR-539 mimics or miR-539 mimics-NC. A model of osteosarcoma subcutaneously transplanted in nude mice was constructed to observe the effect of miRNA-539 on the growth rate of osteosarcoma in vivo.

**Results:**

After transfection of miRNA-539 mimics in osteosarcoma cells 143b and MG63, the proliferation level, migration ability, and invasion ability of the osteosarcoma cells were significantly lower than that in the control group, and the apoptosis level was significantly higher than that in the control group (*P* < 0.01). The dual luciferase reporter confirmed that TRIAP1 was the target of miR-539, and the expression level of TRIAP1 in 143b and MG63 transfected with miRNA-539 mimics was proved to be significantly lower than that in the control group (*P* < 0.01).The western blot showed the expression of genes targeted by TRIAP1 was upregulated when the expression of TRIAP1 was downregulated. In vivo, the osteosarcoma growth rate in the miRNA-539 mimics group was significantly slower than that in the control group (*P* < 0.01).

**Conclusions:**

MiRNA-539 may inhibit the cell proliferation, migration and invasion of osteosarcoma cells and promote the apoptosis of osteosarcoma cells by targeting on TRIAP1.

## Background

Osteosarcoma (OS), occurring frequently in the long bones, is the most common primary bone malignancy in children and adolescents. At present, the diagnosis of osteosarcoma mainly depends on imaging examination, and the best treatment for osteosarcoma is surgical resection combined with adjuvant chemotherapy [1–3]. However, due to the early metastasis and drug resistance of osteosarcoma, the 5-year survival rate of patients is still low. It has been reported that the 5-year survival rate of non-metastatic patients is 65–70 % and that of metastatic patients was 10–20 % [4]. In order to diagnose osteosarcoma as early as possible and to improve the prognosis of patients with osteosarcoma, there is an urgent need to find new diagnostic methods and treatment strategies for osteosarcoma.

MicroRNAs (miRNAs) are short non-coding RNAs whose lengths range between 18 and 25 nucleotides (nt). Accumulating evidence has demonstrated that miRNAs regulate gene expression through degrading mRNAs or suppressing translation by binding to target mRNAs in the 3′-untranslated regions (3′-UTRs) [5, 6]. A lot of studies have demonstrated that miRNAs are related to cellular processes, including cell proliferation, migration, invasion and apoptosis [7–9]. Our previous study have found that miR-539 is downregulated in osteosarcoma cell lines compared with osteoblasts cell lines [10] and miR-539 was also found to be downregulated in other kinds of cancers [11, 12]. However, the effect of miR-539 on osteosarcoma and the mechanism remains unclear. In the current study, we investigated the roles of miR-539 and the underlying mechanism in the regulation of osteosarcoma cell biological behaviours.

TRIAP1 gene, also known as p53CVs, is induced by p53 gene when cells are under low stress [13]. Studies have shown that TRIAP1 is involved in the occurrence of a variety of tumours, such as myeloma, ovarian cancer, and nasopharyngeal carcinoma [14–17]. High expression of TRIAP1 in tumours often indicates poor prognosis for patients [18, 19]. A previous study has reported that the decrease in TRIAP1 expression is related to the enhancement of chemosensitivity in osteosarcoma [20].

In this study, we found that miR-539 inhibited cell proliferation, invasion and migration of osteosarcoma in vitro and in vivo, which may occur mechanistically through targeting TRIAP1. It is expected that this study can provide a new therapeutic target for osteosarcoma patients and may improve diagnosis and therapies for osteosarcoma patients.

## Materials and methods

### Cell culture

The human osteosarcoma cell lines 143b and MG63 and the osteoblast cell line hFOB1.19 were obtained from the China Centre for Type Culture Collection (Wuhan, China). The 143b cells were cultured in RPMI-1640 (Gibco, USA), and the MG63 and hFOB1.19 cells were cultured in DMEM (Gibco, USA), with both media supplemented with 10 % foetal bovine serum (Gibco, Scoresby, Australia) and 1 % penicillin and streptomycin. 143b and MG63 cells were maintained at 37 °C in a cell incubator with 5 % CO_2_ while hFOB cells were maintained at 34 °C in a cell incubator with 5 % CO_2_.

### Cell transfection

miR-539 mimics and negative control miRNA (miRNA NC) were purchased from GenePharma (Shanghai, China). The 143b and MG63 cells were transfected with miR-539 mimics or miRNA NC using Lipofectamine 2000 (Invitrogen) according to the manufacturer’s instructions. The transfection efficiencies were evaluated by reverse transcription-quantitative polymerase chain reaction (RT-qPCR) at 48 h posttransfection. And then we used a lentivirus (Hanbio, Shanghai China) with TRIAP1 overexpression to transfect 143b and MG63 cells in miR-539 mimic group.

### Cell counting kit-8 (CCK-8) assay

Transfected cells were cultured at a density of 2 × 10^3^ transfected cells/well in 96-well plates. At 0, 24, 48, and 72 h after the cells were transfected, 10 µl CCK-8 working solution was added into each well, followed by incubation for 2 h in a cell incubator. Next, the absorbance of each well was determined by an enzyme-linked immunosorbent assay reader (Bio-Rad) at a wavelength of 450 nm. Each experiment was repeated three times.

### Apoptosis assay

Cell apoptosis were evaluated through flow cytometry. At 48 h posttransfection, the cells were collected, washed twice in PBS at 4 °C, resuspended in 500 µl of 1 × binding buffer, and then incubated with an Annexin V/PI double staining kit for 5 min in the dark. After that, the stained cells were examined with a flow cytometer, and the data were analysed using CytExpert 2.0 and Flowjo software (Beckman Counter, USA). Each experiment was repeated three times.

### Wound‐healing assay

A wound-healing assay was used to determined cell migration. The transfected cells were seeded in 6-well plates. The cells were scratched with a 200-µl pipette tip when the cell confluency reached 90 %. The detached cells were removed washing twice with PBS. The medium was replaced with Opti-MEM Reduced-Serum Medium (Gibco). The images of the wound closure were photographed at both 0 and 24 h after initiation of the wound. Each experiment was repeated three times.

### Transwell invasion assay

The invasion ability of cell was evaluated by transwell invasion assay. Transwell chambers were coated with Matrigel (BD, Bioscience). Subsequently, 5 × 10^4^ transfected cells resuspended in 200 µl FBS-free culture medium were placed in the top chamber. Meanwhile, 500 µl medium containing 20 % FBS was placed into the lower chamber. After incubation for 24 h, the cells attached to the upper surface of the membrane were removed with a cotton tip. Thenirdly, the invasive cells attached to the lower surface were fixed with formaldehyde for 10 min and then stained with 0.1 % crystal violet (Sigma-Aldrich). The invasive cells were imaged and counted in five random fields. Each experiment was repeated three times.

### RT-PCR assays

Total RNA was isolated from cells using TRIzol reagent (Invitrogen) according to the manufacturer’s instructions. RNA was reverse transcribed to cDNA using a Prime Script RT Reagent Kit (Toyobo, Japan). SYBR Green qPCR Master Mix (Thermo Fisher Scientific) was used for quantitative real-time PCR. For the determination of miR-539, U6 was used as an endogenous control. For the detection of TRIAP1, GAPDH was used as an endogenous control. The specific primers were as follows: has-miR-539 5′-GGAGAAATTATCCTTGGTGTGT-3′; U6-F 5′-CTCGCTTCGGCAGCACATA-3′; U6-R 5′-CGAATTTGCGTGTCATCCT-3′. The 2^−△△Ct^ equation was used to calculate the relative expression level. Each experiment was repeated 3 times.

### Western blots

Total protein was extracted, and the protein concentration was determined by the bicinchoninic acid method. The cell proteins were separated by SDS-PAGE on an 8–12 % gel and electrotransferred onto polyvinylidene difluoride membranes (EMD Millipore, Billerica, MA, USA). Next, the membrane was placed in 5 % skimmed milk powder for 2 h for blocking. Later, the membranes were washed three times with a Tris-buffered solution with Tween 20 (TBST), after which primary antibodies against TRIAP1, p53, p21, apaf1, and casepase9 (1:1000, Abcam, UK)were added for an overnight incubation at 4 °C. The membranes were washed with TBST again, after which horseradish peroxidase-conjugated IgG was added for another 2 h of incubation. Last, after being washed with TBST three times, the membranes were visualized using an enhanced chemiluminescence kit (Bio-Rad) and a ChemiDoc™ Imaging System (Bio-Rad), and the ImageJ software was used to analyse the optical density of the membranes.

### Bioinformatics prediction and dual luciferase reporter assay

We predicted the putative targets of miR-539 using the online tool, TargetScan (http://www.targetscan.org/).

The dual-luciferase miRNA target expression vector pmirGLO (Promega, Madison, USA) was used to establish luciferase reporter constructs. Putative miR-539-binding sites on the 3′-UTR of TRIAP1 were predicted by bioinformatics analysis. The human wild-type (WT) TRIAP1 3′-UTR containing putative miR-539-binding sites ( 5′-AUUUCUC-3′) was amplified by PCR and cloned into the pmirGLO vector. The human mutant (MUT) of TRIAP1 3′-UTR containing a mutant miR-539-binding site (5′-UAAAGAG-3′) was amplified by PCR and cloned into the pmirGLO vector. Cells were seeded in 96-well plates and cotransfected with wild-type or mutant TRIAP1 3′UTR constructs and miR-539 mimic or negative control. Luciferase activity was measured with a dual-luciferase reporter assay system (Promega). Firefly luciferase activity was normalized against Renilla luciferase activity.

### Establishment of orthotopic osteosarcoma xenograft tumour models

The animal study was approved by the ethical committee of Zhongnan Hospital of Wuhan University.This study was executed in the Animal Laboratory Center, Zhongnan Hospital of Wuhan University. Following previous published methods [21], orthotopic osteosarcoma xenograft tumour models were established successfully. In short, 143b cells were resuspended in 100 µl PBS (the concentration of 143b cells was 10^7^/mL) and injected into the subperiosteum of the left proximal lateral tibia of 4- to 6-week old male BALB/nude mice (about 10^6^ 143b cells were injected into each nude mice). Next, the mice were divided into two groups: the miR-539 agomir (Ribobio, Guangzhou China) treatment and the agomir control group. Ten days after the cells were injected, the volume of the tumours were approximately 100 mm^3^. The 100 µl miR-539 agomir or the agomir control was injected intratumourly every three days. The length and width of the tumours were measured with a digital caliper every day. The tumour volume was calculated with the following equation: tumour volume (mm^3^) = length × width^2^ × 0.5. Digital plain-film X-ray was used to evaluate the orthotopic tumours. After the mice were sacrificed, the tumours were excised and weighed, and the lung tissue was also removed for further analysis. The obtained tissues were fixed with paraformaldehyde, embedded in paraffin, and then stained with H&E. And we observed them by microscope for histological analysis. Embedding, fixation and H&E staining were carried out according to the previous published methods [21].

### Statistical analysis

All experimental data are expressed as the means ± standard error. The statistical analyses of the data were performed using SPSS 17.0 software. All data were analysed using student’s-test or one-way ANOVA. One-way ANOVA was used when more than 2 groups were compared, followed by Bonferroni multiple comparisons post-hoc test. *P* < 0.05 was considered a statistically significant difference.

## Results

### Restoration of miR-539 inhibited cell proliferation and promoted cell apoptosis in 143b and MG63 cells

**Fig. 1:**
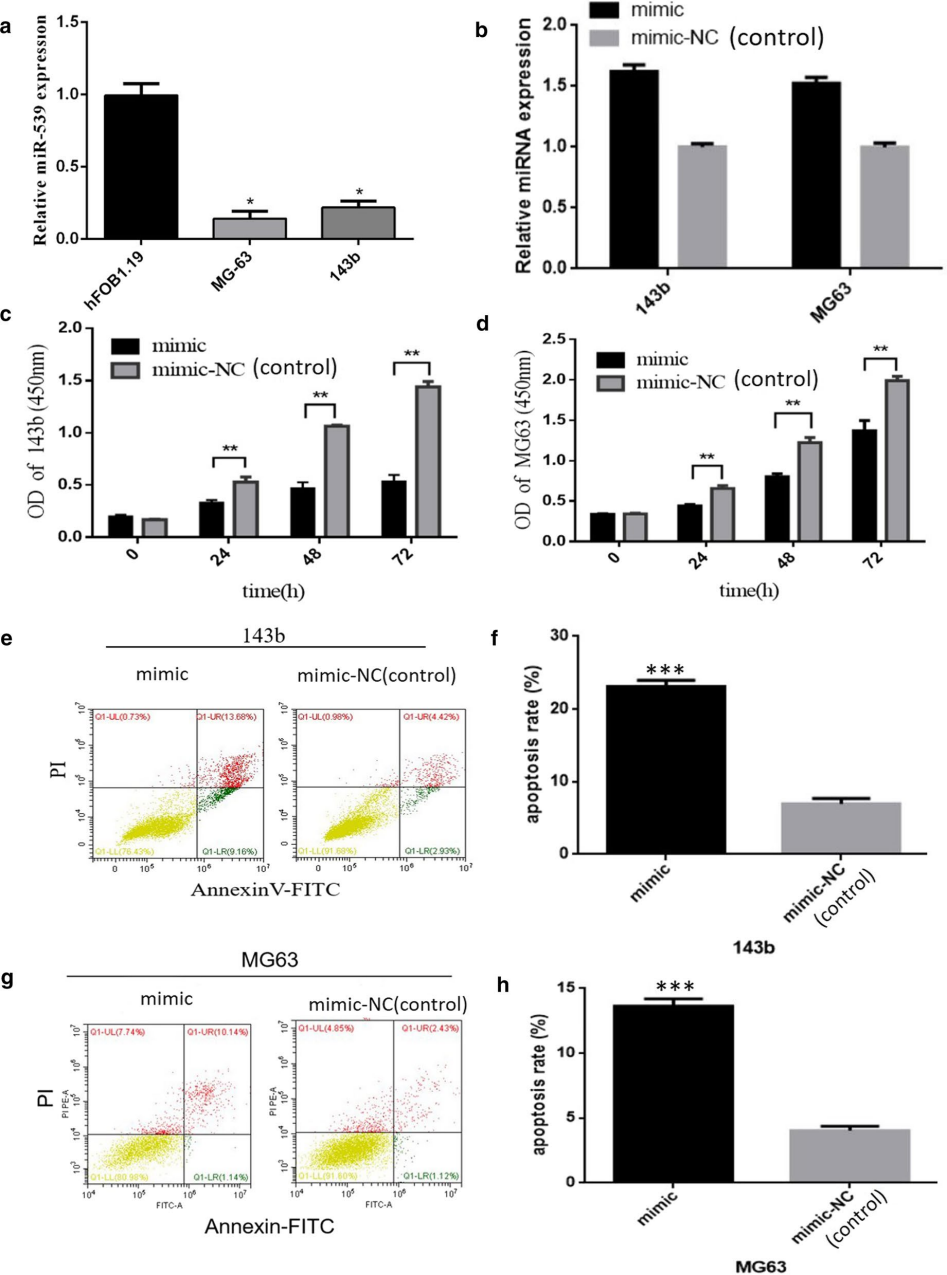
Restoration of miR-539 inhibited cell proliferation and promoted cell apoptosis in 143b and MG63 cells. **a** The data show that the expression of miR-539 was much lower in osteosarcoma cell lines (143b, MG63) than in an osteoblastic cell line. **b** The data show that the expression of miR-539 in the miR-539 mimics group (both 143b and MG63) was much higher than that in the miR-539 mimic-NC group and that the difference between the two groups was statistically significant (*P* < 0.01). **c** The cell proliferation of 143b osteosarcoma cells. **d** The cell proliferation of MG63 osteosarcoma cells. The pictures in **c** and **d** show that the cell proliferation in the miR-539 mimic groups was much lower than that in the miR-539 mimic-NC groups (*P* < 0.01). **e–f** Apoptosis of osteosarcoma 143b cells. **g–h** Apoptosis of osteosarcoma MG63 cells. All of the above pictures show that the miR-539 mimic groups had higher rates of apoptosis than the miR-539 mimic-NC groups in both the 143b and MG63 osteosarcoma cell lines (*P* < 0.01)

To explore the role of miR-539 in osterosarcoma, we first evaluated the expression of miR-539 in two osterosarcoma cell lines using RT-PCR. The data demonstrated that the expression of miR-539 was much lower in 143b and MG63 osteosarcoma cells than in hFOB1.19 osteoblastic cells (Fig. 1a). Then, we restored the miR-539 level by transiently transfecting with miR-539 mimics, and the transfection efficiencies were confirmed by RT-qPCR (Fig. 1b). A CCK-8 assay was used to assess the effect of miR-539 on osteosarcoma cell proliferation. The data showed that the optical density values were lower in the miR-539 mimics group when compared with the control group 24, 48, and 72 h after posttransfection in 143b and MG63 cells (Fig. 1c, d). Furthermore, as observed through flow cytometry, higher rates of apoptosis were observed in the miR-539 mimics group than in the control group in the 143b (Fig. 1e, f) and MG63 (Fig. 1g, h) cells. Above all, these phenomena suggested that miR-539 inhibited cell proliferation and promoted cell apoptosis in 143b and MG63 cells.

### The overexpression of miR-539 suppressed migration and invasion abilities in 143b and MG63 cells

**Fig. 2:**
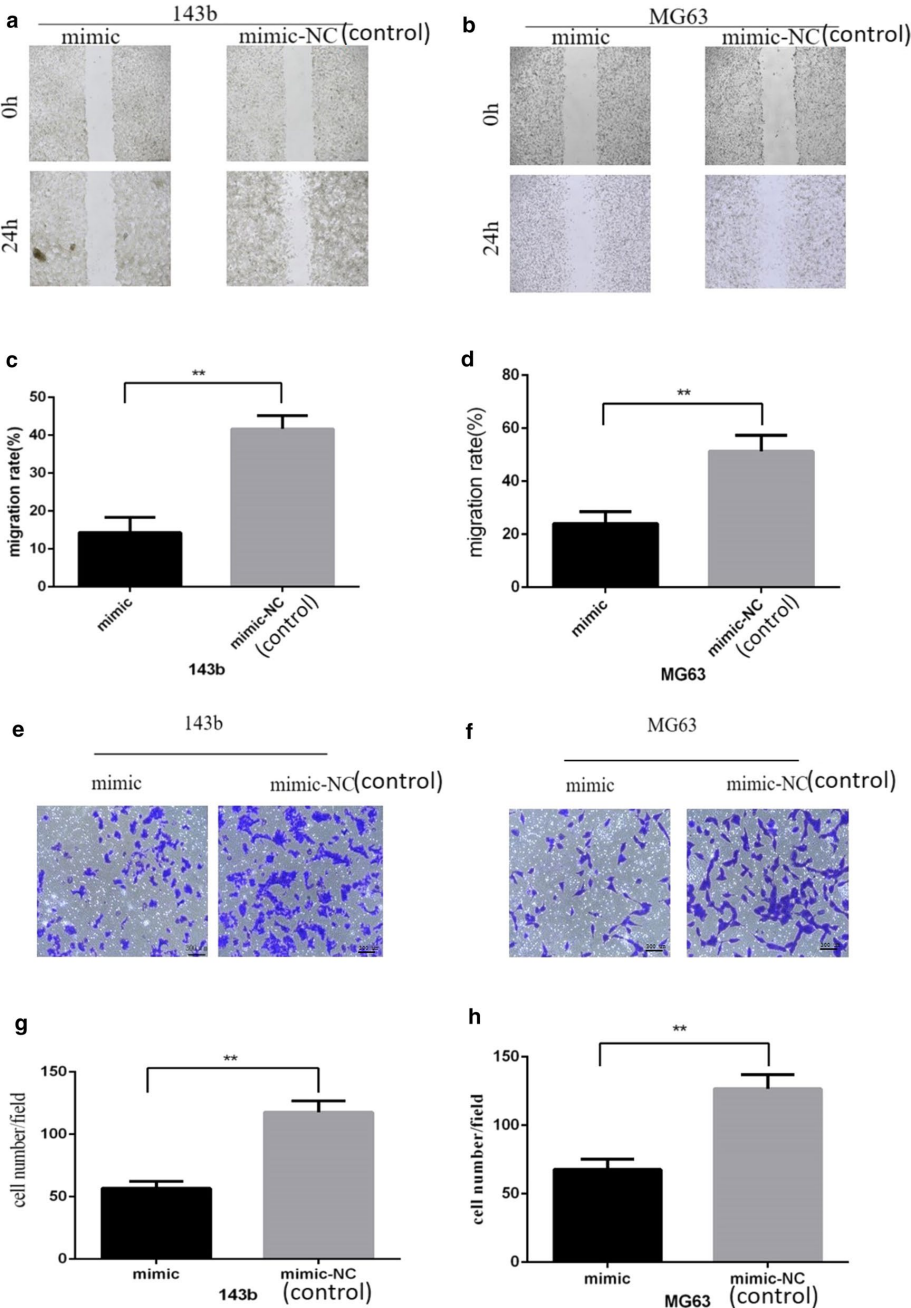
The overexpression of miR-539 suppressed the migration and invasion abilities of 143b and MG63 cells. Effect of overexpression of miR-539 on migration and invasion of the osteosarcoma 143b and MG63 cells. **a**, **b** The migration abilities of 143b cells. **c**, **d** The migration abilities of MG63 cells. **e**, **f** The invasion ability of 143b cells. **g**, **h** The invasion ability of MG63 cells. The data in **a** through **d** indicates that the migration abilities of 143b and MG63 cells transfected with miR-539 mimics decreased significantly compared with the control group. The data in **e** through **h** show that the invasion ability of 143b and MG63 cells transfected with miR-539 mimics decreased significantly compared with the control group. All of the above data demonstrate that miR-539 might inhibit the ability of migration and invasion of osteosarcoma cell lines

To evaluate the effect of miR-539 on migration and invasion abilities in osteosarcoma cells, we carried out a wound-healing assay and a transwell invasion assay with the miR-539 mimics group and the control group. The wound-healing assay revealed that the migration abilities of 143b and MG63 cells transfected with miR-539 mimics much decreased compared with the control group (Fig. 2a–d) (*P* < 0.01). At the same time, after miR-539 was overexpressed in 143b and MG63 cells, the cells showed lower invasion ability than the control group (Fig. 2e–h) (*P* < 0.01). Above all, these results demonstrated that miR-539 inhibited the migration and invasion of the 143b and MG63 osteosarcoma cell lines.

### TRIAP1 is targeted by miR-539

**Fig. 3:**
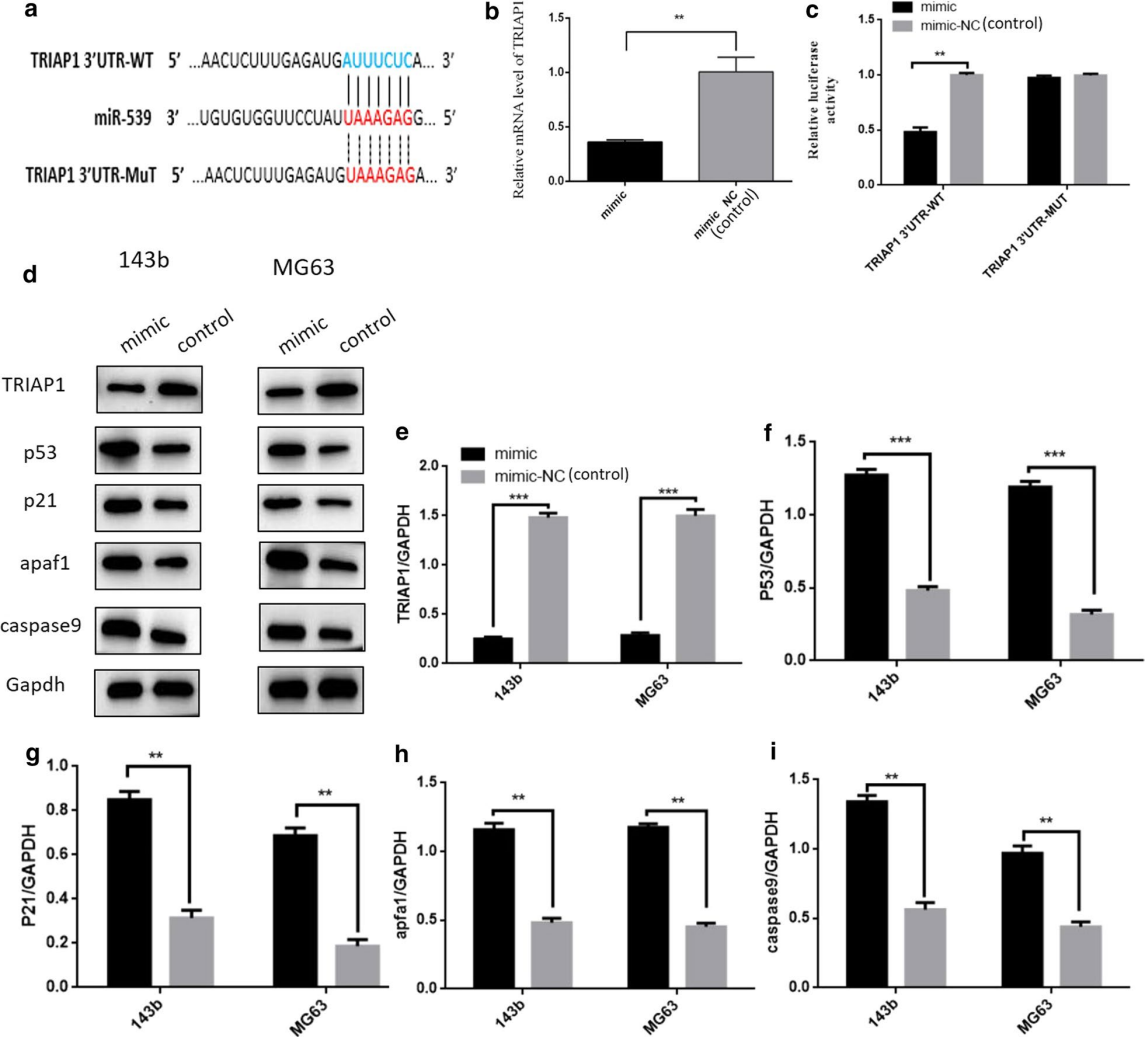
TRIAP1 was targeted by miR-539. Effect of miR-539 on the target gene (TRIAP1) expression. **a** The predicted binding sites of miR-539 and TRIAP1. **b** Expression level of TRIAP1 after transfection of miR-539 mimics or miR-539 mimics NC. The expression of TRIAP1 in 143b was significantly decreased after transfection with miR-539, indicating that miR-539 might inhibit the proliferation and growth of osteosarcoma cells by regulating the expression of the TRIAP1 gene. **c–h** The results of western blotting show that the expression of p53, p21, apaf1 and caspase9 was increased while the expression of TRIAP1 was decreased in 143b and MG63 cells transfected with miR-539 mimics

To further investigate the mechanism of miR-539 inhibition on the osterosarcoma cell lines, we used the online bioinformatics software TargetScan to analyse the underlying target genes of miR-539 and found that TRIAP1 was the target of miR-539 (Fig. 3a). TRIAP1 is known to be able to inhibit apoptosis by regulating the apoptosis pathway and downregulating the expression of its downstream genes, such as p53, p21, apaf1 and caspase9. To confirm the accuracy of this online prediction, we assessed the expression of the TRIAP1 gene and those genes regulated by TRIAP1, such as p53, p21, apaf1 and caspase9. We assessed the expression level of TRIAP1 with the method of qPCR 48 h after transfection of miR-539 mimics in 143b, and the results showed that the expression level of TRIAP1 was lower in the miR-539 mimics group than that in the control group (Fig. 3b, *P* < 0.01). We also used a dual-luciferase reporter system containing a wild-type or mutant 3′-UTR of TRIAP1 to verify whether miR-539 directly interacts with the 3′-UTR of TRIAP1. The 143B cells were co-transfected with miR-539 and either pmirGLO-TRIAP1-3′-UTR-wt or pmirGLO-TRIAP1-3′-UTR-mut (Fig. 3a). The result showed that the transfection with miR-539 suppressed the luciferase activity of pmirGLO-TRIAP1-3′-UTR-wt but did not affect the luciferase activity of the pmirGLO reporter carrying the mutant TRIAP1-3′-UTR (Fig. 3c, *P* < 0.01). The results of western blot also showed that the expression level of TRIAP1 was decreased in the 143b and MG63 cells transfected with miR-539 mimics when compared with that in the control group. Interestingly, the results of the western blot also showed that the expression of p53, p21, apaf1 and caspase9 was increased when the expression of TRIAP1 decreased in the miR-539 mimics group (Fig. 3d–i).

### Overexpression of TRIAP1 rescued the inhibitory effect of miR-539 on 143B and MG63 cells

**Fig. 4:**
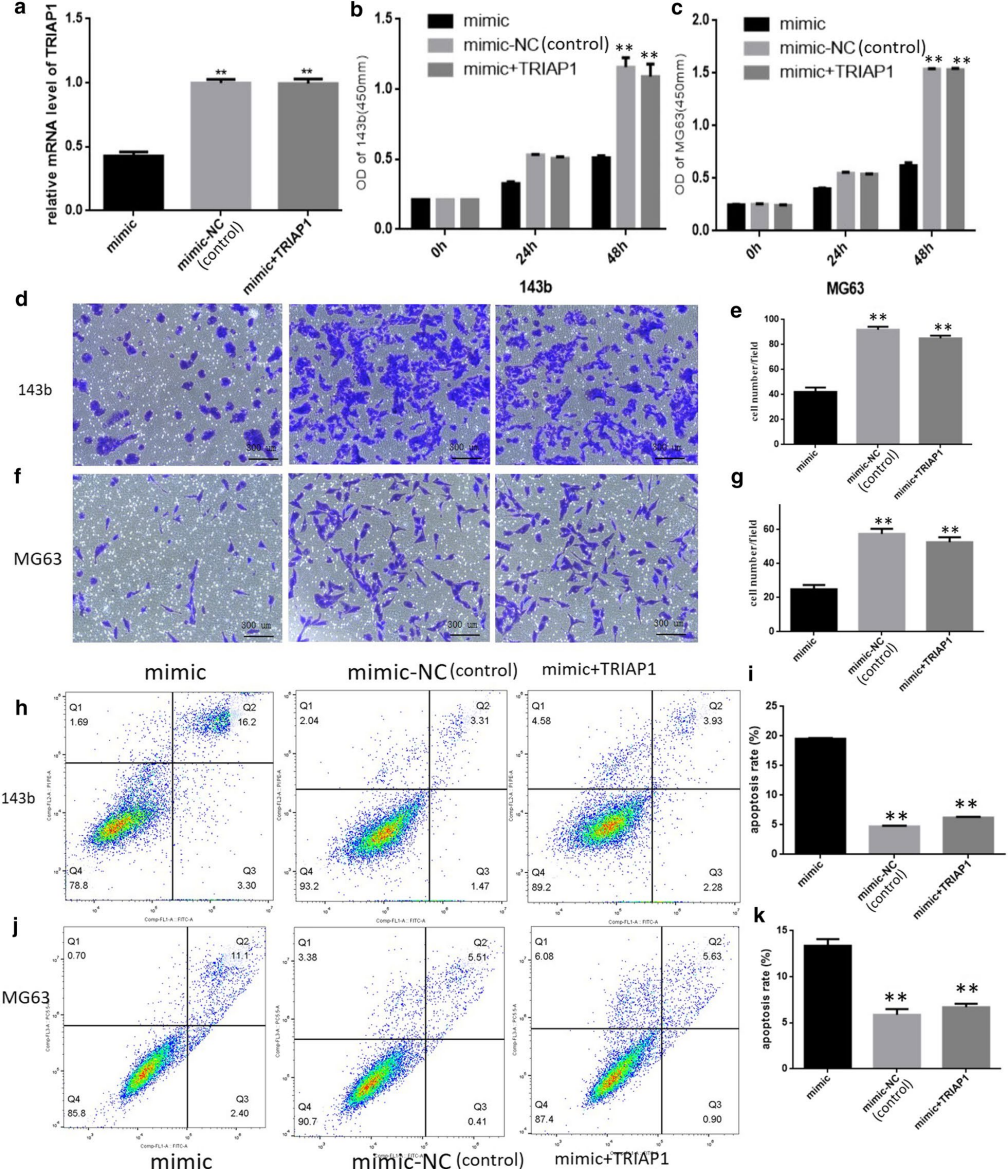
Overexpression of TRIAP1 rescued the inhibitory effect of miR-539 for 143B and MG63 cells. **a** The expression of TRIAP1 was lower than that in mimic-NC group and mimic + TRIAP1 group. **b**, **c** The results of the CCK-8 assay show that the optical density values were lower in the miR-539 mimics group when compared with the miR-539 mimic-NC group and the miR-539 + TRIAP1 overexpression group at 72 h posttransfection in 143b and MG63 cells. **d–g** The results of the transwell assay show that the invasion ability of 143b and MG63 cells in the miR-539 group was much lower than in the miR-539 mimic-NC group and the miR-539 + TRIAP1 overexpression group. **h–k** The results of flow cytometry analysis show that the rates of apoptosis were higher in the miR-539 mimics group than in the miR-539 mimic-NC group and the miR-539 + TRIAP1 overexpression group. All of the data show that there was no obvious difference between the miR-539 mimic-NC group and the miR-539 + TRIAP1 overexpression group

To further demonstrate the tumour suppressor function of miR-539 through targeting TRIAP1, we overexpressed TRIAP1 for 143b and MG63 cells in the miR-539 mimic group, and we named this group as group “*mimic + TRIAP1*”. We can see that in mimic group, the expression of TRIAP1 was lower than that in mimic-NC group and mimic + TRIAP1 group (Fig. 4a). As shown in Fig. 4b, c, the results of a CCK-8 assay showed that the optical density values of 143b and MG63 cells were lower in the miR-539 mimic group than that in miR-539 mimic-NC group and the mimic + TRIAP1 group at 72 h, but there was no obvious difference between the mimic-NC group and the mimic + TRIAP1 group. In other words, overexpression of TRIAP1 counterbalanced the tumour suppressor effect of the miR-539 mimics on cell proliferation in 143B and MG63 cells. Furthermore, the results of the transwell invasion assay showed that the invasion ability of 143b and MG63 cells in the miR-539 mimic group was obviously lower than that in the miR-539 mimic-NC group and the mimic + TRIAP1 group, but there was no obvious difference between the mimic-NC group and the mimic + TRIAP1 group (Fig. 4d–g). Similarly, by the method of flow cytometry, higher rates of apoptosis could be observed in the miR-539 mimic group when compared with that in the miR-539 mimic-NC group and the mimic + TRIAP1 group, but there was no obvious difference between the mimic-NC group and the mimic + TRIAP1 group (Fig. 4h–k). In conclusion, miR-539 is able to inhibit proliferation and invasion and promote apoptosis in 143b and MG63 cells, and overexpression of TRIAP1 can counterbalance these effects. These results further indicated that miR-539 regulated osteosarcoma cells by inhibiting the expression of the TRIAP1 gene.

### Tumour growth and metastasis were inhibited by overexpression of miR-539 in nude mice

**Fig. 5:**
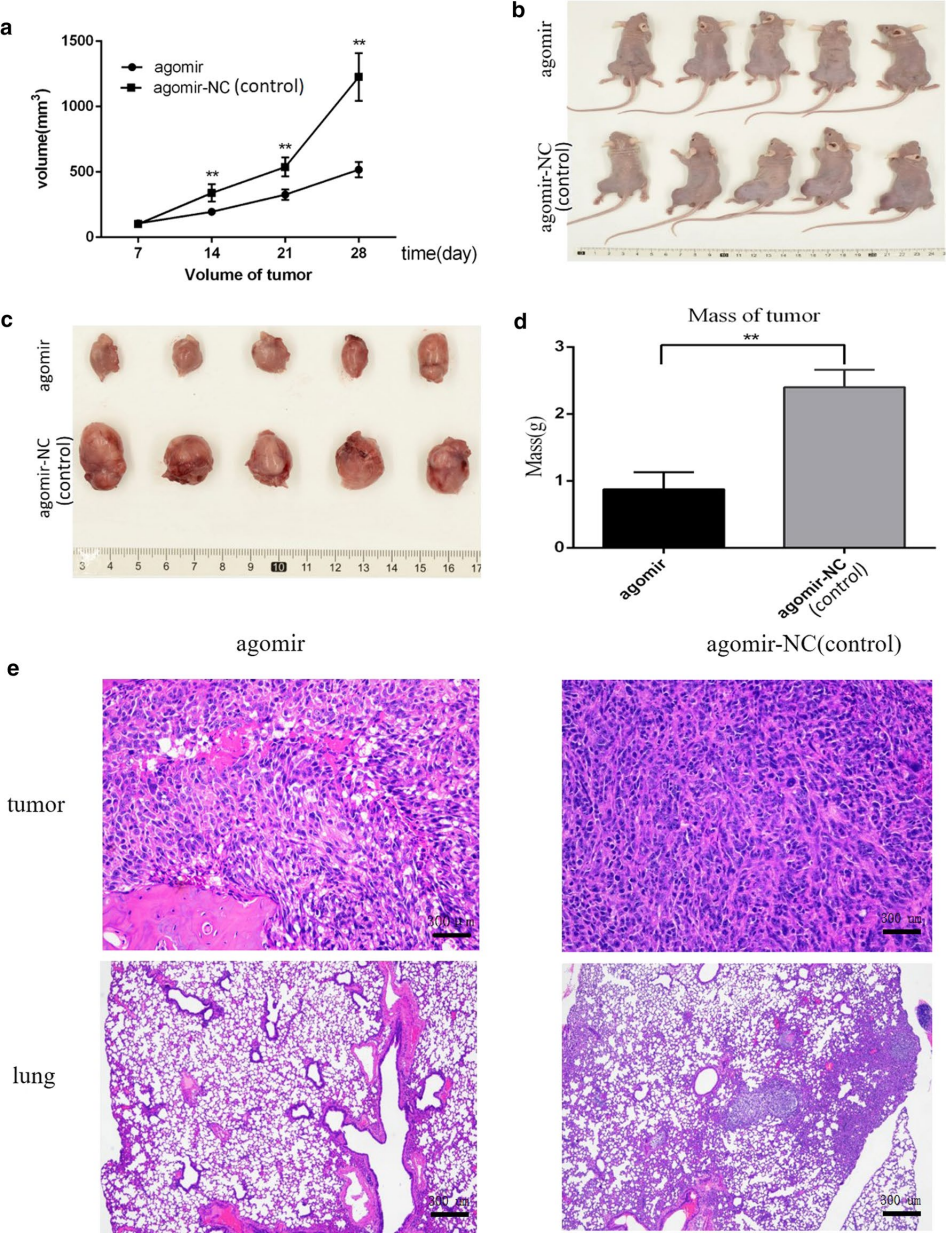
Effect of miR-539 on the growth rate of osteosarcoma in vivo. **a–d** The change in tumour volume in the miR-539 agomir group and the miR-539 agomir NC group. The data show that the growth rate of the osteosarcoma cells in the miR-539 agomir group was much lower than that in the agomir NC group (*P* < 0.01). The necrotic areas in the osteosarcoma cells in the miR-539 agomir group were larger than those in the agomir NC group and in the miR-539 agomir group, and the number of lung metastasis was much lower than in the control group (**e**). All of the above data indicate that miR-539 played a role in inhibiting osteosarcoma cells growth and metastasis in vivo

In this experiment, we established orthotopic osteosarcoma xenograft tumour models to investigate the suppression of miR-539 on tumour growth and metastasis in vivo. Ten days after the injection of 143b cells into the subperiosteum of left proximal lateral tibia, the mice were treated with a miR-539 agomir or agomir-NC by intratumoural injection. As shown in Fig. 5a–d, the tumour volume of the miR-539 agomir treatment group was much smaller than that in the agomir NC group. After the mice were sacrificed, the tumours and lungs were excised and stained with H&E for histological analysis. The necrotic areas in the osteosarcoma cells in the miR-539 agomir group were larger, and the smaller necrosis areas in the osteosarcoma cells could be observed in the agomir control group (Fig. 5e). The number of lung nodules was used to evaluate tumour metastasis. In the miR-539 agomir group, the number of lung metastasis was much lower than that in the control group (Fig. 5e). Above all, these results showed that the miR-539 agomir inhibited osteosarcoma cell growth and metastasis in vivo.

## Discussion

As a non-coding RNA, miRNAs cannot encode for proteins but can regulate gene expression in cells. Increasing investigation indicated that miRNAs have a pivotal role in tumour occurrence and metastasis [22, 23]. The aberrant expression of miRNAs is an important sign in the initiation and progression of cancer [24, 25]. Hence, miRNAs are expected to be used as biomarkers in early diagnosis. In addition, miRNAs have a good chance of becoming new targets for therapeutic uses. In this study, we found that the inhibitory role of miR-539 in the initiation and progression of osteosarcoma was achieved by targeting TRIAP1.

The dysregulation of miR-539 has been reported in different types of cancer. For example, miR-539, which has an effect against HCC development and metastasis, has been proven to be reduced in hepatocellular carcinoma (HCC)[26]. With the ability to enhance chemosensitivity to cisplatin in non-small cell lung cancer (NSCLC), miR-539 has also been proven to be downregulated in NSCLC [27]. Gastric cancer has low a miR-539 expression, and miR-539 has been shown to be able to inhibit the proliferation and migration of gastric cancer cells [28]. Furthermore, the expression of miR-539 is low in thyroid cancer [29] and breast cancer [30]. And previous literature has reported that miR-539 can suppress osteosarcoma cell invision and migration in vitro by targeting Matrix metallopeptidase-8 [31].These investigations indicated that miR-539 may play a pivotal role in various human tumours, including osterosarcoma. In this present study, we found that miR-539 was downregulated in osteosarcoma cell lines, which was similar to our previous microarray study [10]. Moreover, the restoration of miR-539 expression in osteosarcoma cell lines inhibited cell proliferation, migration and invasion and induced cell apoptosis. In addition, in an in vivo experiment, we treated orthotopic osteosarcoma xenograft tumour models with a miR-539 agomir, and the results showed the growth and metastasis of osteosarcoma were inhibited. These observations suggested that miR-539 plays a tumour suppressor role to regulate osteosarcoma growth and metastasis.

miRNAs play an important role in regulating cell progression by targeting genes. Therefore, finding the target gene of miR-539 helps us to understand the mechanism of miR-539 suppression of osteosarcoma progression. Recent studies have found several target genes for miR-539, including MAP2K1 [26], DCLK1 [27], SRY-box5 [28], CARMA1 [29], epidermal growth factor receptor [30] and DIXDC1 [32]. We selected TRIAP1 as a putative target gene of miR-539 through bioinformatics analysis. In the study, we confirmed that miR-539 could reduce TRIAP1 mRNA and protein expression in in vitro experiments. These results proved that miR-539 exerted a tumour suppressor function to impair osteosarcoma cell proliferation and invasion through targeting TRIAP1.

TRIAP1, previously known as P53CSV, is induced significantly by p53 when cells have a low level of genotoxic stress, and TRIAP1 interacts with Hsp70 to block formation of the Apaf-1/procaspase9 complex, promoting cell survival during tumour progression [33]. Fook-Alves et al. [13] revealed that the stable silencing of TRIAP1 induced late apoptosis through the Apaf-1/procaspase9 pathway in RPMI8226 cell line. Potting et al. [16] found that TRIAP1/PRELI complexes protect cells from apoptosis upon intrinsic and extrinsic stimulation. In addition, relationships between several miRNAs and TRIAP1 have been found. For instance, a previous study revealed that TRIAP1 was the direct target gene of miR-18a in ovarian cancer [15]. Another study reported a new miR-320b/TRIAP1 pathway and that miR-320b acts as a posttranscriptional regulator of TRIAP1 expression in nasopharyngeal carcinoma [14]. These studies suggest that TRIAP1 may also play an important role in the development of osteosarcoma.

In this study, we investigated the effect of miRNA-539 on the growth of osteosarcoma in vivo and in vitro. For in vitro experiments, we mainly carried out cell-based experiments using the osteosarcoma 143b and MG63 cell lines to verify the role of miRNA-539. For in vivo experiments, we constructed an orthotopic transplantation model of osteosarcoma in nude mice to observe the inhibitory effect of miRNA-539 on the growth rate of osteosarcoma. We found that the expression of miRNA-539 in the 143b and MG63 cell lines was relatively low, so we chose to observe the effect of miRNA-539 on the occurrence and development of osteosarcoma. Then, we upregulated the level of miRNA-539 by transfection and carried out a CCK-8 assay, wound-healing assay and transwell assay, in which we ultimately found that miRNA-539 inhibited the proliferation, migration and invasion of 143b and MG63 osteosarcoma cells. Flow cytometry results showed that miRNA-539 could induce apoptosis in the 143b and MG63 cells. In addition, we found that TRIAP1 was the target of miRNA-539, as the expression level of TRIAP1 in osteosarcoma cells transfected with miRNA-539 mimics significantly decreased. The results of the western blotting also indicated that the expression of TRIAP1 decreased while the expression of the genes targeted and inhibited by TRIAP1 was increased. Finally, we verified the inhibitory effect of miRNA-539 on the growth of osteosarcoma in vivo. The results showed that the tumour weight of the miRNA-539 agomir group was significantly smaller than that of the control group, and the X-ray evaluation showed that the destruction degree of the tibial bone in the miRNA-539 agomir group was significantly weaker than that in the agomir NC group and that the tumour volume growth curve showed that the tumour growth rate of the miRNA-539 agomir group was significantly slower than that of the agomir NC group. After H&E staining, it was observed that the number of osteosarcoma cells in the miRNA-539 agomir mimics group was significantly lower than that in the control group, that the cell heteromorphism in the miRNA-539 agomir mimics group was relatively small, and the mitotic images in the miRNA-539 agomir mimics group were fewer. All these results indicate that miRNA-539 can inhibit the growth of osteosarcoma by decreasing the expression of TRIAP1.

In conclusion, the expression of miRNA-539 in the 143b and MG63 osteosarcoma cell lines was lower than that in the normal human osteoblast hFOB1.19 cell line. Overexpression of miR-539 could inhibit the proliferation, migration and invasion of the two osteosarcoma cell lines. In addition, we found that miRNA-539 could inhibit the growth of osteosarcoma in vivo in nude mice. At the same time, we found that the expression level of TRIAP1 decreased when miR-539 was overexpressed. Hence, we have concluded that miR-539 can inhibit the growth of osterosarcoma by downregulating the expression of TRIAP1 and that the possible molecular mechanism involves miR-539 binding to the 3′ UTR of TRIAP1 and destroying the apoptosis inhibition of TRIAP1 by activating its downstream genes, such as p53, p21, apaf1 and caspase9. However, the TRIAP1 gene is regulated by the TP53 gene [13], miR-1301 [20], miR-18a [15], and miR-320b [14], and miR-539 can regulate the expression of SP1 [34], TWIST1 [35], and DCLK1 [27]. In this study, we only investigated the effect of miR-539 on TRIAP1 and osterosarcoma, but more details need to be examined and further research should be undertaken.

## Data Availability

The datasets used and/or analyzed during the current study are available from the corresponding author on reasonable request. And all the data has been submitted as supplemental material to editors.

## Abbreviations

miR-539: microRNA-539
OS: Osteosarcoma
UTRs: Untranslated regions
DMEM: Dulbecco’s modified Eagle’s medium
